# Improvement of migraine symptoms with a proprietary supplement containing riboflavin, magnesium and Q10: a randomized, placebo-controlled, double-blind, multicenter trial

**DOI:** 10.1186/s10194-015-0516-6

**Published:** 2015-04-03

**Authors:** Charly Gaul, Hans-Christoph Diener, Ulrich Danesch, on behalf of the Migravent® Study Group

**Affiliations:** Migraine and Headache Clinic, Königstein im Taunus, Germany; Department of Neurology, University Hospital Essen, Essen, Germany; Weber & Weber GmbH & Co.KG, Clinical Research, Inning, Germany

**Keywords:** Migraine, Prevention, Nutritional Supplement

## Abstract

**Background:**

Non-medical, non-pharmacological and pharmacological treatments are recommended for the prevention of migraine. The purpose of this randomized double-blind placebo controlled, multicenter trial was to evaluate the efficacy of a proprietary nutritional supplement containing a fixed combination of magnesium, riboflavin and Q10 as prophylactic treatment for migraine.

**Methods:**

130 adult migraineurs (age 18 – 65 years) with ≥ three migraine attacks per month were randomized into two treatment groups: dietary supplementation or placebo in a double-blind fashion. The treatment period was 3 months following a 4 week baseline period without prophylactic treatment. Patients were assessed before randomization and at the end of the 3-month-treatment-phase for days with migraine, migraine pain, burden of disease (HIT-6) and subjective evaluation of efficacy.

**Results:**

Migraine days per month declined from 6.2 days during the baseline period to 4.4 days at the end of the treatment with the supplement and from 6.2.days to 5.2 days in the placebo group (p = 0.23 compared to placebo). The intensity of migraine pain was significantly reduced in the supplement group compared to placebo (p = 0.03). The sum score of the HIT-6 questionnaire was reduced by 4.8 points from 61.9 to 57.1 compared to 2 points in the placebo-group (p = 0.01). The evaluation of efficacy by the patient was better in the supplementation group compared to placebo (p = 0.01).

**Conclusions:**

Treatment with a proprietary supplement containing magnesium, riboflavin and Q10 (Migravent® in Germany, Dolovent® in USA) had an impact on migraine frequency which showed a trend towards statistical significance. Migraine symptoms and burden of disease, however, were statistically significantly reduced compared to placebo in patients with migraine attacks.

## Background

Migraine is a functional disorder of the brain. The pathophysiology of migraine involves many different mechanisms including modulation of central and peripheral pain structures and release of vasoactive peptides. Patients typically experience episodes of headaches, mostly throbbing, unilateral and severe which vary within and among patients. Headaches are frequently accompanied by other symptoms like nausea, phonophobia and/or photophobia [1].

Acute attacks are treated with different analgesics or triptans. Only a minority of migraineurs take preventive medication to decrease the frequency, duration and severity of migraine attacks. Prophylactic drug treatment of migraine should be considered when the quality of life is severely impaired, when two or more attacks occur per month, when migraine attacks do not respond to acute drug treatment or in case of intolerance to or side effects of acute treatment [2].

The guidelines of the German Headache and Migraine Society and the American Academy of Neurology recommend primarily beta-blockers, antiepileptics (topiramate or valproic acid), and antidepressants (e.g. amitriptyline) for migraine prevention [3,4]. Vitamin B2 (riboflavin), magnesium and coenzyme Q10 are alternatives to drugs and appeal to patients with a desire for more natural treatment. In addition, micronutrients are seen by the patients as a "mild" form of treatment with no or minor side effects [5,6].

Studies revealed decreased levels of the micronutrients riboflavin, magnesium and coenzyme in plasma and in the brain of migraine patients [7-9]. A deficit of these nutrients could play a role in the pathophysiology of migraine. Mitochondrial dysfunction is associated with migraine [10,11]. Riboflavin, magnesium and coenzyme Q10 play an important role in the production of energy in the mitochondria [12]. Magnesium is needed in various physiological processes which influence the pathophysiology of migraine (vasoconstriction, platelet inhibition, secretion of serotonin). Magnesium is also needed as a co-factor for proper functioning of the ATP-synthase which produces ATP. Furthermore, Mg is the physiological antagonist at the NMDA-channel which is involved in the regulation of neuronal excitability. Riboflavin is a precursor for flavin-mononucleotide (FMN) and flavin-adenine-dinucleotide (FAD). Both are essential components of complex I and complex II responsible for electron-transport in the mitochondrial membrane. Coenzyme Q10 is a vitamin-like compound which can be synthesized by the body from phenylalanine and tyrosine. Coenzyme Q10 is needed for all cellular processes requiring energy. Coenzyme Q10 is an electron-carrier, transferring electrons from complex I/complex II to cytochrome C. Based on these observations, it seems plausible that a substitution of these micronutrients in migraine patients might be able to prevent or reduce the intensity of migraine attacks. Migraine treatment with a nutritional supplement might be of benefit for patients with recurrent migraine who cannot tolerate chemical drugs due to side effects or contra-indications due to concomitant diseases.

The commercially available food supplement Migravent® in Germany (Dolovent® in the USA) contains riboflavin, magnesium and coenzyme in high doses along with low-dose multi-vitamins for support of general health. This supplement has already been tested in an open clinical study with 31 migraine patients in Germany [13]. The present trial was conducted to prove the efficacy of Migravent®/Dolovent® compared to placebo in a larger number of patients and under randomized, double-blind and multicenter conditions.

## Methods

### Participants and recruitment

Otherwise healthy adults aged 18 to 65 years of either sex were recruited by neurologists practicing in Germany. All participants had migraine with and without aura diagnosed according to the IHS-criteria ICHD-II 1.1 und 1.2 [14]. The age at onset of migraine was less than 50 years of age and diagnosis of migraine was at least one year before study entry. The participants were required to have at least 3 migraine attacks per month in the last 3 months before recruitment and not more than 10 headache days. Patients were excluded if they used migraine prevention (drugs, nutritional supplements or psychotherapy) as well as antipsychotic or antidepressant medication during the last 3 months prior to study entry and throughout the study. Patients with medication overuse were excluded. Patients who had failed to respond to more than 2 different prophylactic agents in the past and patients resistant to all acute migraine drugs were not included.

The study was approved by the Institutional Review Board for each center. All participants gave written informed consent. The study was conducted according to the ethics principles of the Declaration of Helsinki. The trial was registered in the German Clinical Trial Register DRKS00004565.

### Interventions

The investigational nutritional product (INP) is a dietary food for special medical purposes (according to EU regulations) containing 400 mg riboflavin (vitamin B2), 600 mg magnesium, 150 mg coenzyme Q10 along with a multivitamin/trace elements combination per 4 capsules (Migravent®, Dolovent®). The amount of additional multivitamin/trace elements per 4 capsules is as follows: 750 μg vitamin A, 200 mg vitamin C, 134 mg vitamin E, 5 mg thiamin, 20 mg niacin, 5 mg vitamin B6, 6 μg vitamin B12, 400 μg folic acid, 5 μg vitamin D, 10 mg pantothenic acid, 165 μg biotin, 0.8 mg iron, 5 mg zinc, 2 mg manganese, 0.5 mg copper, 30 μg chromium, 60 μg molybdenum, 50 μg selenium, 5 mg bioflavonoides. Placebo capsules indistinguishable from verum were used as control. Patients were instructed to take two capsules orally in the morning and two capsules in the evening for 3 months. Treatments for other conditions which may have an effect on migraine prevention were not allowed. These were mainly beta-blockers (e.g. propranolol, bisoprolol, metoprolol), calcium-antagonists (e.g. flunarizine), antiepileptics (e.g. topiramate, valproate), antidepressants (e.g. amitryptiline), supplements containing petasites (butterbur) or tanacetum (feverfew), magnesium, riboflavin or coenzyme Q10 in doses above 50 mg. Non-medical/non-nutritional treatment for migraine prevention like acupuncture or psychotherapy were also not permitted. However, participants were allowed to treat migraine attacks with their usual rescue pain medication and anti-emetics.

### Study design

The study was conducted as a randomized, placebo-controlled, parallel-arm, double-blind, prospective multi-center study. After screening, patients underwent a one-month baseline period without treatment to verify that they had more than 3 migraine attacks but not more than 10 days with migraine or non-migraine headaches. The baseline phase also served as a baseline for the evaluation of efficacy parameters. Before entering the baseline phase patients had to meet inclusion and exclusion criteria (see Participants and recruitment). Demographic data, concomitant medication, medical history, migraine diagnosis as well as previous migraine preventive measures were documented by the investigator. Following the baseline phase and provided no inclusion/exclusion criteria were violated, eligible patients were randomized in double-blind fashion to verum or to placebo (1:1). In this follow-up visit patients were also asked to fill in an HIT-6 questionnaire. Randomization was done by computer and randomization lists were prepared. Randomization was done by blocks of four per center. The investigator sequentially allocated the random numbers to patients, starting from the lowest number. A blockwise randomization was used. The sequential order was verified by fax sent to a blinded person at the sponsor and from entries in the screening logs. Both investigator and patient were blinded to the treatments. Treatment with either verum or placebo was for 3 months. Migraine parameters and intake of the investigational products were recorded daily by the patients throughout the baseline and the treatment phase in an electronic diary accessed online via the internet. Compliance (documentation, intake of investigational products) was monitored regularly by the investigator and delegates of the sponsor. Patients were immediately contacted by the investigator if regular documentation was missing for more than a week. At the follow-up visit at the end of the treatment the patients again had to fill in an HIT-6 questionnaire and they were asked to evaluate the tolerability and efficacy of the treatment from their view. Concomitant medication and occurrence of adverse events were checked at each follow-up visit. Compliance was assessed by a pill count of the returned investigational product.

### Efficacy parameters

The primary efficacy endpoint was defined as days with migraine as recorded in the online diary by the patient. Secondary endpoints were maximal pain of migraine headaches as recorded in the online diary, migraine burden as assessed through the HIT-6 questionnaire [15] and subjective patient evaluation of efficacy.

Days with migraine and migraine pain intensity were compared between the one-month baseline period and the last month of the 3-month treatment. A migraine day was defined as a day with at least 4 hours of migraine pain or a day with migraine pain and concomitant intake of pain medication. For each migraine day, pain intensity was rated as mild, moderate or severe by the patient. The HIT-6 questionnaire (headache impact test), which measures the impact of headache on a patient´s life, was filled out by the patient at the start of the treatment (randomization) and at the end of the 3-month treatment. This validated questionnaire consists of 6 questions, each question or item has the following response options: never (6 points), rare (8 points), sometimes (10 points), very often (11 points) and always (13 points) [14]. Headache impact on this scale ranges from 36 (no headache) to 78 (very severe headache). All checked points are added for the analysis. Efficacy of the treatment as a subjective evaluation by each patient was recorded at the end of the study as very good, good, moderate or poor.

### Statistical methods

The sample size estimation was performed with the Test of the Ratio of Two Poisson Means module of PASS 11 software (NCSS, LLC. Kaysville, Utah, USA). A reduction by at least 50% in the number of migraine days should be achieved in the verum group because this was considered a clinically relevant improvement and a 30% reduction in the placebo group was assumed as the worst case scenario since placebo effects of around 30% have been seen in trials. Based on these assumptions, the estimated sample size without compensation for drop-out is 39.2 ≈ 40 patients per treatment group, i.e. 80 patients in total. The sample size was increased to 104 patients in total to compensate for overdispersion and a 15% drop-out rate.

The statistical analysis was based on the ICH Topic E9 Note for guidance on statistical principles for clinical trials (CPMP/ICH/363/96). A detailed description of the statistical evaluation was provided in a Statistical Analysis Plan (SAP).

The primary endpoint migraine days was compared statistically in a confirmatory test approach on superiority of Migravent® compared to placebo. The respective statistical test was performed using the generalized linear model in the following form: it was assumed that the number of migraine days during the last month of the 3-month treatment period can be described by Poisson distributions. The migraine day rate depends on treatment and disease severity at baseline, which is defined as the number of migraine days during the one-month baseline period. This was modeled through a Poisson regression with covariate “number of migraine days during the one-month baseline period” and with the factors “treatment” and “center”. The null hypothesis to be tested was whether the rate ratio for treatment (ρ = λ_Placebo_/λ_Verum_) is smaller or equal to 1 (i.e. log rate ratio is smaller or equal to 0). The type-I error rate was set to α = 0.025 (one-sided). Confounding factors were not controlled for.

The maximal intensity of migraine pain per migraine day during the last month of the 3-month treatment was compared between treatment groups by an ANCOVA with the maximal intensity of migraine pain during the baseline period as baseline covariate.

The burden of disease measured by HIT-6 sum scores for the end of treatment was compared between treatment groups by an ANCOVA with the burden of disease sum scores for the start of treatment as baseline covariate.

Subjective evaluation of efficacy by the patient at the end of therapy was displayed by default descriptive statistics for continuous data as well as for categorical data. The treatment groups were compared by van-Elteren’s test.

## Results

### Recruitment

**Figure 1:**
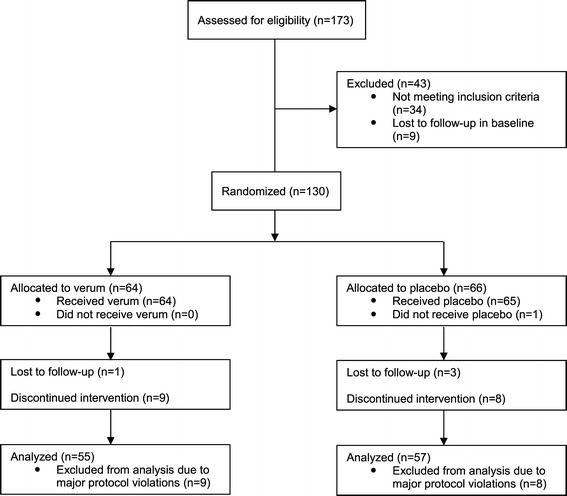
**Consort diagram showing recruitment and flow of participants through trial.**

173 migraine patients fulfilled the inclusion- and exclusion criteria and were enrolled in the baseline phase. The enrollment took place in 12 neurological centers in Germany from October 2012 to November 2013. The baseline phase consisted of a 30-day period without prophylactic migraine treatment. 34 patients could not be randomized into the treatment groups because they failed inclusion and fulfilled exclusion criteria after baseline; of the 34 patients, 4 had no migraine attack at all, 21 had between 1 and 2 migraine attacks per month and 9 had more than 10 days with migraine. Nine patients were lost during the baseline phase, mostly due to non-compliance with the use of the online diary. The number of patients with prior migraine prevention was slightly higher in the placebo group (40 vs 36), however, 3 vs 1 patients in the verum group had used more than 3 migraine preventions in the past. The same, to a slightly more extent, is observed regarding medical history and concomitant medications. Of the 130 patients randomized, one patient provided no efficacy data since he did not open the diary during the treatment phase and did not show up for the final visit. Due to major protocol violations, 9 patients in the active-group and 8 patients in the placebo-group were excluded from efficacy analysis (Figure 1).

The baseline characteristics of all patients included in the efficacy analysis are described in Table 1. There was no significant difference in baseline parameters. 37 patients (64.9%) in the placebo group had migraines without aura compared to 28 patients (50.9%) in the active group. The number of patients without any prophylactic migraine treatments in their medical history was similar in both groups. However, the number of patients with up to 3 different prophylactic treatments in the past was higher in the placebo group (35.0%) than in the active group (21.8%). Number of concomitant diseases and medications was higher in the placebo group.

**Table 1:** **Baseline characteristics of evaluable participants**

**Characteristic**	**Verum**	**Placebo**
	**N = 55**	**N = 57**
Female n (%)	48 (87.3)	49 (86.0)
Age y (SD)	40.4 (13.39)	36.4 (11.14)
BMI (SD)	23.16 (3.57)	23.17 (3.55)
Migraine type		
With aura n (%)	22 (40.0)	16 (28.1)
Without aura n (%)	28 (50.9)	37 (64.9)
Previous migraine prevention		
Participants with no previous preventions n (%)	40 (72.7)	36 (63.2)
Participants with 1-3 previous preventions n (%)	12 (21.8)	20 (35.0)
Participants with more than 3 previous preventions n (%)	3 (5.4)	1 (1.8)
Medical history, diseases n (%)	43 (42.1)	59 (57.8)
Concomitant medication n (%)	24 (36.3)	42 (63.6)

n denotes numbers, events or medication, respectively.

### Reduction of migraine days

Active treatment was able to reduce the number of days with migraine from 6.2 days in the baseline-phase to 4.4 days after 3 month of treatment by 1.8 days (Table 2). However, this reduction of migraine days compared to placebo was not statistically significant (p = 0.23).

**Table 2:** **Reduction of migraine days**

**Days with migraine (SD)**	**Verum**	**Placebo**	**P value**
	**N = 55**	**N = 57**	
Baseline	6.2 (1.95)	6.5 (1.78)	-
Treatment 1st month	5.0 (3.39)	5.7 (3.03)	0.37
Treatment 2nd month	4.8 (3.29)	5.5 (3.01)	0.39
Treatment 3rd month	4.4 (2.99)	5.2 (3.22)	0.23

### Maximal pain intensity per migraine day

Verum reduced the mean maximal pain intensity of a migraine day based on a 3-point-scale by 0.24 points at the end of the 3-month treatment. This reduction was statistically significant compared to placebo (0.06 points, p = 0.03) (Table 3). The percentage of patients with severe pain was lower and the percentage of patients with mild pain at the end of the 3-month treatment phase was higher in the active group compared to placebo.

**Table 3:** **Reduction of maximal pain per migraine day**

**Intensity (SD)**	**Verum**	**Placebo**	**P value**
	**N = 55**	**N = 57**	
Baseline	2.71 (0.458)	2.70 (0.533)	-
Treatment 1st month	2.55 (0.503)	2.63 (0.620)	0.17
Treatment 2nd month	2.44 (0.572)	2.53 (0.630)	0.53
Treatment 3rd month	2.47 (0.639)	2.64 (0.520)	0.03
**Patients (%)**	**Verum**	**Placebo**	**P value**
	**N = 55**	**N = 57**	
Baseline			-
- mild	0 (0)	2 (3.5)	
- moderate	16 (29.1)	13 (22.8)	
- severe	39 (70.9)	42 (73.7)	
Treatment 3rd month			0.03
- mild	4 (7.3)	1 (1.8)	
- moderate	20 (36.4)	18 (31.6)	
- severe	29 (52.7)	37 (64.9)	

### HIT-6 Questionnaire (headache impact test)

Verum reduced the sum score of the HIT-6 questionnaire by 4.8 points (from 61.9 points at baseline to 57.1 points at the end of the 3-month treatment). This reduction was statistically significant compared to placebo (p = 0.01). The reduction of HIT-6 sum scores in the placebo group was 2 points (from 61.9 points to 59.9).

### Evaluation of efficacy by the patient

At the end of the 3-month treatment, the efficacy as evaluated by the patient was statistically significantly superior compared to placebo (p = 0.01) (Table 4). No patient in the placebo group rated the efficacy as very good, whereas 18% of the patients treated with verum rated the efficacy as very good. Nearly half of the patients in the placebo group rated the efficacy as poor (43.9%) compared to only 29.1% in the active treatment group.

**Table 4:** **Evaluation of efficacy by patient**

	**Verum**	**Placebo**	**P value**
	**N = 55**	**N = 57**	
Mean (SD)	2.6 (1.09)	3,2 (0.81)	0.01
very good n (%)	10 (18.2)	0 (0)	
good n (%)	16 (29.1)	14 (24.6)	
moderate n (%)	13 (23.6)	18 (31.6)	
poor n (%)	16 (29.1)	25 (43.9)	

### Safety

Adverse events were classified by System Organ Class (SOC) and Preferred Term (PT) of the MedDRA-coding system. The safety population consisted of n = 63 patients in the verum group as well as in the placebo group. No serious adverse events (SAE) were observed in this trial. The incidence of adverse events (AE) was higher under active treatment (34 AE in 21 (33.3%) of 63 patients) compared to placebo (9 AE in 7 (11.1%) of 63 patients). All adverse events whose causal relationship to the study treatment was assessed by the investigator as at least possibly related were classified as adverse reactions (AR). The incidence of adverse reactions was higher under active treatment (24 AR in 15 (23.8%) of 63 patients) compared to (3 AR in 3 (4.8%) of 63 patients). The two most frequent adverse reactions were gastrointestinal disorders (verum: 10 AR in 8 (12.7%) of 63 patients; placebo: 2 AR in 2 (3.2%) of 63 patients) mainly diarrhea and chromaturia (verum: 8 AR in 8 (12.7%) of 63 patients; placebo: 0 AR in 0 (0%) patients).

The majority of adverse reactions observed under active treatment were completely recovered before the end of the study (21 (87.5%) of 24 AR). While most adverse reactions (13 AR (54.2%)) did not lead to any action regarding the study treatment, 3 (12.5%) adverse reactions led to dose change, 6 (25%) adverse reactions to permanent discontinuation of the active treatment and 2 (8.3%) adverse reactions to another treatment.

## Discussion

Drugs like metoprolol, propranolol, flunarizine, valproic acid or topiramate have been shown in clinical trials to be effective in reducing migraine symptoms when administered as prophylactic agents in episodic migraine [16-22]. All of these drugs have potential side effects, sometimes of severe nature. For this reason many patients look for a natural preventive treatment of migraine. In fact, some clinical trials have been performed with magnesium [23-29], riboflavin (vitamin B2) [30-35] or ubiquinone (ubichinon, coenzyme Q10) [36-38] mostly as single agents. One RCT used a combination of magnesium, riboflavin and the botanical feverfew [31] which revealed no advantage over the control group (intake of 25 mg riboflavin), most likely due to the fact that 25% - 38% of the patients in the verum and control group took concomitant migraine prophylaxis during the study. To the best of our knowledge, there is no report of a randomized, double-blind and controlled trial with a triple combination of magnesium, riboflavin and coenzyme Q10. It is interesting to note, that a pharmacogenomic study demonstrated the importance of the mitochondrial genetic background on response to riboflavin [39]. This underlines the role of the mitochondrion in migraine and the potential role of magnesium, coenzyme Q10 and riboflavin in alleviating migraine symptoms. It can also explain why certain patients are non-responders.

Baseline values were comparable between the treatment groups. The number of patients who used migraine prevention before study entry was slightly higher in the placebo group. The numbers are too small to be significant and to have had an impact on the primary endpoint. The same, to a slightly more extent is observed regarding medical history and concomitant medications. Since patients were allocated in a strict randomized fashion to both treatment arms this observation might be pure chance. It is not justified to suggest that patients in one group who seem to be slightly more ill than patients in the other group would show a difference in the prevention of migraine.

In this clinical trial, a combination of three natural nutritional substances, magnesium, riboflavin and Q10, was tested against placebo in the treatment of migraine in adult patients. Treatment for 3 months with this proprietary nutritional supplement was able to reduce the number of days with migraine by almost 2 days (1.8), which is considered to be a clinically relevant reduction. The reduction by placebo was 1.3 days. However, the reduction in migraine days was not statistically significant.

A very similar reduction was achieved in a randomized, placebo-controlled study (MIGR-003) with topiramate [19]. Migraine days were reduced by 1.8 days by topiramate 100 mg/day and by 1.1 days by placebo. This result was statistically significant (p = 0.026). However, the number of patients in the 100 mg topiramate arm was 139 compared to 55 in the arm of this trial with the nutritional supplement containing a fixed combination of magnesium, riboflavin and Q10. This suggests that the trial might have been underpowered with regard to the primary endpoint migraine days. The fact that the 200 mg topiramate arm in the MIGR-003 study did not reach statistical significance even with 143 patients (probably due to many early drop-outs) suggests that 55 patients are simply not enough to show statistical significance of any treatment in reducing migraine days.

Otherwise, the secondary endpoints that were analyzed in addition to the reduction of migraine days in this trial demonstrated a statistically significant benefit of the triple combination compared to placebo. Patients treated with verum had a statistically significantly greater reduction in the maximally experienced pain per migraine day compared to placebo (p = 0.03). In the verum group, 70.9% of the patients reported severe migraine pain prior to treatment. At the end of the treatment, only 52.7% had severe pain, 7.3% had mild pain. In the placebo group, 73.7% patients had severe pain in the baseline period, 64.9% patients had severe pain at the end of treatment and only 1.8% patients had mild pain.

The beneficial efficacy of verum was also shown by a statistically significant reduction in the score of the headache impact test questionnaire HIT-6 (p = 0.01). The sum score of the questionnaire was reduced in the active group after 3 months of treatment by a mean of 4.8 points. A primary care population of migraine patients was analyzed in the publication by Smelt. A within-person minimal important change (MIC) was established between -2.5 and -5.5 points depending on the statistical approach [40]. The within-person MIC is defined as the smallest change in the score which patients perceive as important. The reduction of -4.8 points in this trial is therefore a clinically relevant improvement which shows statistical significance compared to placebo. The HIT-6 questionnaire is a scale with 5 response options. The response options never (6 points), rarely (8 points) and always (13 points) are not represented in the study population because those patients were excluded from participation based on the inclusion and exclusion criteria. Therefore, sometimes (10 points) and very often (11 points) are left as the only possible answers for the study population, with only a 1-point difference between them. This 1-point difference in the questionnaire corresponds to a relevant difference in medical terms or in terms of disease burden/headache impact. A 4.8 point reduction translates into nearly 5 items (questions in the questionnaire) being improved from very often to sometimes. Smelt also established a between-group minimally important difference (MID) of -1.5 points [40]. Similar to the within-person MIC, the between-group MID is the smallest difference between scores of groups of patients that is considered important. The difference between the placebo group and the verum group was -2.8 points in this trial.

In agreement with the above result, the evaluation of efficacy of the preventive treatment was better for verum than for placebo. The difference was statistically significant (p = 0.01). 18.2% of the patients in the active group rated the efficacy as very good, none in the placebo group. Nearly 50% (47.3%) of the patients rated the efficacy “very good” or “good” which was twice as many as in the placebo group. Also, the number of patients rating efficacy as poor was higher in the placebo group.

These results indicate that the study preparation might have had an impact on the frequency of migraine days and improved clinically relevant prespecified secondary endpoints such as pain intensity, headache impact on life (HIT-6) and subjective evaluation of effectiveness.

The triple combination had a favorable adverse event profile. Adverse events usually observed with drugs like weight gain, depression, tiredness or dizziness were not observed.

A shortcoming is the possibility of unblinding patients in the verum group due to chromaturia. However, every patient was told at the beginning of the trial that a discoloration of the urin might appear in order to rule out that chromaturia would be associated with verum only. The only way to avoid this would have been to add riboflavin to placebo.

The strength of this study is the prospective, double-blind and placebo-controlled design. The study was powered to show a possible difference for the primary endpoint. The study used validated endpoints.

## Conclusions

A fixed combination in a daily dose of 600 mg magnesium, 400 mg riboflavin and 150 mg Q10 in a proprietary nutritional supplement including also various low-dose multivitamins did not show statistically significant efficacy in the reduction of migraine days probably due to being underpowered. It did, however, prove to be superior for several secondary outcomes in the treatment of migraine. After 3 months of treatment with the supplement, a reduction of migraine pain and burden of disease was seen. Patients rated the efficacy of the treatment significantly superior to placebo. Adverse events associated with the supplement were mainly abdominal discomfort and diarrhea due to high amounts of magnesium. There were no serious adverse events reported in this trial.
